# Examining the gaps in perinatal mental health care: A qualitative study of the perceptions of perinatal service providers in Canada

**DOI:** 10.3389/fgwh.2023.1027409

**Published:** 2023-03-15

**Authors:** Christina DeRoche, Amanda Hooykaas, Christine Ou, Jaime Charlebois, Krista King

**Affiliations:** ^1^ Research Centre, Canadore College of Applied Arts and Technology, North Bay, ON, Canada; ^2^ Department of Geography, Environment and Geomatics, University of Guelph, Guelph, ON, Canada; ^3^ School of Nursing, Institute of Aging and Lifelong Health, University of Victoria, Victoria, BC, Canada; ^4^ Community Mental Health Service, Orillia Soldiers Memorial Hospital, Orillia, ON, Canada; ^5^ Faculty of Nursing, Memorial University of Newfoundland, St. John's, NL, Canada

**Keywords:** perinatal mental health disorders, national policy, Canadian context, marginalized population experiences, qualitative study

## Abstract

In Canada, access to perinatal mental health services is disparate across districts, regions, provinces, and territories. Questions remain as to how gaps in service are being experienced by Canadian service providers and clinicians. This paper examines three key questions: 1) What are the experiences of care providers with respect to the screening, identifying, and managing perinatal mental health disorders? 2) What gaps in perinatal mental health care have been identified? and 3) What approaches have been taken by providers, communities, and regions in addressing the needs of their populations? To address these questions, 435 participants from across Canada were surveyed using an online survey constructed by the research members of the CPMHC. A qualitative analysis of the data revealed three key themes: groups marginalized by the current perinatal mental health system, gaps and supports identified by communities; and systemic and policy issues. From these three themes we have identified the key components of changes required in the national approach to perinatal mental health disorders. We identify key resources that could be utilized to create policy change and provide recommendations for change.

## 1. Introduction

While pregnancy, childbirth, and parenting are often seen as times of excitement, the period between conception to one-year postpartum is also associated with increased risk for onset and relapse of mental health conditions for women (1). During this time, the focus of care tends to be on the infant rather than screening for maternal mental health conditions (2). Without routine standardized screening, three-quarters of women meeting Diagnostic and Statistical Manual of Mental Disorders, Fifth Edition (DSM-V) criteria for depressive and anxiety disorders are not identified (3) and only 10% of women requiring mental health care receives care (4). Perhaps more concerning is that even if diagnosed, only 15% of women received evidence-based treatment (4). Barriers to accessing perinatal mental health services are significant and often those who may require this support the most are unable to receive it.

Perinatal Mental Health Disorders (PMHD) are characterized by distressing feelings that range in intensity from mild to severe during pregnancy (prenatal) and throughout the first year after birth (postpartum). PMHDs are serious, largely underdiagnosed issues that may persist if untreated (4, 5). Long et al. (6) indicated that risk factors for developing PMHDs include a history of depression/anxiety, low marital satisfaction, intimate partner violence, lack of social support, and isolation. Increased PMHDs severity are often associated with impaired functioning, especially in relation to a woman's ability to care for her infant and the formation of secure infant attachment, which may in turn be associated with poorer social, cognitive, and behavioural outcomes in the child (7). It is therefore imperative that families experiencing psychosocial problems and mental health conditions in the perinatal period are identified early to receive timely support and care. Approximately 1 in 5 women experience PMHD in Canada (8). This has increased to 1 in 3 women for depression and 1 in 2 women for anxiety during the COVID-19 pandemic in Canada (9). Moreover, appropriate treatment of PMHD is important: suicide is a leading cause of death during the perinatal period and accounts for 5%–20% of maternal deaths (10).

Robust evidence suggests that psychological and psychosocial interventions for PMHD are effective and cost-efficient (1, 11). Although the gold standard of evidence-based treatment include cognitive behavioral therapy and behavioral-interpersonal therapy, many women face tremendous difficulty accessing support, high out-of-pocket costs, and long wait times for treatment (12). Further, many mental health clinicians have some training in CBT and would be capable of providing some support for those individuals screened as at-risk for PMHD, however, most women are not screened or are not heard by healthcare professionals (please see Statement of Inclusion) (13).

Resources are disparate across Canada (2, 14). Recent perinatal research suggests that integrated interventions following comprehensive assessment are essential for holistic perinatal care, but few have been developed (1); however, not many women are being screened for PMHD to begin with (13). Approximately 87% of health care providers surveyed stated they were not mandated to provide screening for PMHD and over 50% did not have specialized training for this (2). Within the Canadian context, each province has its own approach to PMHD care and screening and there have been a few exemplars/guidelines created, but no national approach or policy exists.

### 1.1. COVID-19 experiences and impact

The COVID-19 Pandemic has significantly impacted the experiences of many new parents, and literature highlights that the impact has increased the need for national approaches to PMHD across the board. For example, Basu et al. (15) conducted the first of its kind, global study exploring the impact of COVID-19 on parental experiences in the postpartum period. They aimed to document the prevalence of posttraumatic stress, anxiety, and depression symptoms as well as loneliness in their study from over 64 countries. They found that women not only worried about their own health and infant's health in the postpartum period, their anxiety was also exacerbated by fear of COVID-19 with 43 to 51% of women experiencing anxiety because of COVID-19 (15). Overall, they found that women were self-reporting increased symptoms of postpartum depression and anxiety during the pandemic. These results have been replicated by others such as Bertholot et al. (16) who compared a cohort of women pre- and mid-pandemic, that with higher rates of depression and anxiety in the postpartum period at mid-pandemic. Canada has lagged behind other countries in developing and implementing a national perinatal mental health strategy that would help address these elevated rates. Other nations such as Australia, the United Kingdom, and the United States have implemented salient components of national approaches to addressing PMHD.

The purpose of this study was to determine the gaps in perinatal mental health care landscape in Canada from health care providers' perspective. In this paper, we closely examine the textual responses of clinicians and providers, to answer the following research questions:

1. What are the experiences of care providers with respect to the screening, identifying, and managing of PMHD?
2. What gaps in perinatal mental health care have been identified?
3. What unique approaches have been taken by providers, communities, and regions in addressing the needs of their populations?

## 2. Materials and methods

### 2.1. Survey design

The Qualtrics survey used in this study was constructed by researchers associated with the Canadian Perinatal Mental Health Collaborative (CPMHC) with the purpose of examining perinatal care providers' perspectives on perinatal mental health care and current standards of care for parents in Canada. The study was approved by the University of Calgary Conjoint Health Research Ethics Board. The survey contained questions about demographics, professional training and roles, and questions about provider practices and perspectives around screening, management of, and referrals for PMHD, and accessibility of care using a combination of open- and close-ended questions. This survey contained 37 questions in total. Some of the qualitative questions included descriptions of practitioner experiences in the field. Survey items were developed by a small group of CPMHC members using an iterative feedback process. The quantitative data is reported elsewhere (14). The survey was offered in both official languages of Canada (English and French).

#### 2.1.1 Participant recruitment and procedures

Individuals were eligible if they identified as a perinatal professional (e.g., physician, midwife, nurse, psychologists, doula, social worker, physiotherapist, or naturopath). Respondents were recruited using social media, email listservs of perinatal service providers, and through snowball sampling. All respondents consented to participate.

This paper reports the findings of respondents' explanations of their responses to questions such as “Do you believe persons from diverse backgrounds encounter any barriers to accessing perinatal services?” and “Have your perinatal patients identified increased difficulty accessing services during the global pandemic? Please list barriers that have affected your practice.” The last question on this survey asked respondents if they had anything else they would like to share about their experiences supporting people through perinatal mental health challenges; this question garnered the most rich, qualitative data used in this analysis.

A thematic network analysis of open-ended responses was completed. Within this survey, the team outlined several different areas important in understanding the approach that providers took in screening, diagnosing, and referring their clients by drawing from theoretical and empirical knowledge. Because the authors are immersed in perinatal mental health practice and research, engaging in reflexivity during the process of thematic analysis was important. Reflexivity refers to acknowledging the researcher's own primary biases and reflecting on how this might impact the interpretation and analysis of the results. The two first authors independently reviewed and analyzed the data and then convened to develop the themes while engaging in reflexive discussions (17). The authors drew from the contextual responses of respondents to create the theoretical understandings of the gaps and needs presenting given the types of questions asked.

Using a selective coding process, responses were coded into six major areas: policy, screening, treatment, follow-up, community initiatives, and training (18). These areas align with the objectives of the CPMHC in the assessment, development, and proposal of policy across Canada, at both the provincial and federal level. The codes were determined by the pre-established research questions. Once collapsed and coded into these five major areas (policy, screening, treatment, community initiatives, and training), researchers began to review each coded area against one another's work, ensuring validity and reliability in the approach to coding and the understanding of the respondent's words. From these five categories, responses were then further reduced to three major themes based on respondents' emergent needs and critical concerns (1) Persons marginalized by the current PMH system; 2) Gaps and Supports Identified by Communities; and 3) Policy and Larger Systematic Gaps and Concerns. Noting that identified concerns can be categorized differently, it was critical in our approach to thematic analysis that the researchers practiced reflexivity in reducing these to three main areas of needs and concern, while being careful to ensure that all responses were captured accurately and fully.

## 3. Results

### 3.1. Respondent characteristics

The survey received responses from 435 respondents from across Canada. Respondents were predominantly female (95.6%), with 41.5% selecting the category of being 31 to 40 years of age. Provinces with the greatest number of respondents included Ontario (50.9%), British Columbia (19.2%), and Quebec (11.8%). Twelve percent of respondents identified as BIPOC. Respondents included physicians (22.8%), nurses (17.7%), midwives (16.1%), naturopathic doctors (13.4%), doulas (12%), social workers (9.4%), therapists (13.2%), psychologists (4.4%), and a small number of allied care professionals such as physiotherapists (4.8%).

The analysis of data using this methodology yielded the following major themes for respondents: 1) groups of people that are marginalized by the current perinatal mental health care system and the barriers to care that they face; 2) gaps and supports identified by community-led approaches and solutions; and 3) policy or systematic approaches and/or issues. The following discussion of results follows these themes as they relate to the research questions.

### 3.2. Theme one: persons marginalized by the current PMH system

Respondents identified many groups of individuals who were marginalized by the current perinatal mental health care system in Canada. It was notable that several respondents articulated that those who were not marginalized by the current system included those who were white, of higher socioeconomic means (e.g., had extended health insurance), and had mild-to-moderate anxiety or depression. This indicated that persons who did not fit this narrow description were likely to experience problems with attaining proper perinatal mental health care.

Many groups of individuals were identified as having limited access to perinatal mental health care or were at risk of not receiving adequate mental health care. These included racialized persons and persons identifying as 2SLGBTQIA + . Respondents echoed how persons of color were likely to experience problems with access to culturally safe care. One respondent stated, “*Racialized and Indigenous people [are] less likely to speak out because babies are apprehended at an alarming rate in these communities.”* Several respondents spoke about how services are not oriented for gender and other 2SLGBTQIA + persons and families. One respondent described how persons such as transgendered parents, “*experience assumptions, homophobia, and misunderstandings… they are tired of explaining (over and over again) their family situation…”* while another respondent described, “*there are not enough options for the gender diverse community.”* Larger systemic issues arise with the appropriate language and background also; for example, one respondent was blunt in her response in pointing out these systematic gaps: “*Cis-heteronormative language is a big red flag for lack of inclusivity. Very little specialized OHIP (Ontario Health Insurance Plan, a government run health insurance plan)-covered support in languages other than English, very few OHIP-covered BIPOC therapists and psychiatrists.”*

Current services were described to be ethnocentric, lacking in culturally appropriate options (e.g., for Indigenous families) with many care providers being predominantly white and many lacking in cultural sensitivity. For example, one respondent stated that: “*Do not forget about Indigenous perinatal mental health as they are referred to us for obstetrical care, but we are unsure of what to do after they receive a diagnosis.”* In this instance, the participant noted the lack of culturally appropriate pathways for those parents identifying as Indigenous. This clearly points to large gaps in our understanding of culturally relevant treatment and intervention options for a significant portion of the Canadian population. Another respondent stated that there was a*“ lack of Indigenous-specific services for perinatal mood issues.”*

Current models of mental health care and psychiatry were also noted to be rooted in Eurocentric paradigms. Services in languages other than English were also noted by many to be nearly non-existent. For example, one respondent stated: “*Services for uninsured patients (who may be more likely to need it), also, a racialized client in our practice experienced racism at the hospital*.

Racialized and Indigenous clients were reported by respondents when accessing care to have been subjected to racism several ways. For example, certain ethnic groups were described to have experienced discrimination, lack of warmth from health care providers, and not offered services or continuing care. This experience was shared by other racialized community members as well, as another respondent described, “*accessing services is already incredibly difficult for the [South Asian] community… many are not offered services or continuing care.” S*everal respondents commented on how BIPOC women were at greater risk of receiving poor quality care and experiencing perinatal complications or having access to none whatsoever.

Some solutions were put forward by respondents, for example:

*“Suggestions I have included is directing funding towards childcare; parental leave; culturally and racially specific mental health supports (i.e. Anti-racist approach that recognizes excess burden that BIPOC experience in obstetric and perinatal health systems); increasing knowledge of trauma informed care among obstetric and perinatal health providers.”*

Other providers aimed at doing their best with the human resources that they had: “*We try to provide diverse and ethnocultural services. My team is primarily Caucasian women. I have one therapist who is part of the LGTBQ community, and one with native heritage.”*

Other minority populations such as those living in rural and remote communities expressed gaps within the system. For example, some respondents mentioned that transportation and services for those in the rural communities is a barrier and presents inequitable conditions for families in rural areas. One participant stated: “*We live in a remote northern community and access to specialized professionals is challenging.”* Another stated that “*there are very few mental health providers with specialized training and often remote or disadvantaged clients cannot obtain support due to finances or transportation.”* Overall living in more Northern communities where rural and remote settings are numerous presented with challenges:

*“Relatively remote communities with limited number of professionals are normal. Often the quality of professionals locally feels lower than that of larger centers where there would be a variety to choose from.”*

This indicates that considerations need to be made for those living within these contexts and recruitment and retention strategies for specializing services is of the utmost priority. Access to mental health care for all individuals cannot be taken for granted. Despite the systematic failings, some communities, especially those with higher proportions of BIPOC and rural/remote populations, devised their own resources to help meet needs as best they could.

### 3.3. Theme two: gaps and supports identified within communities

Many individuals identified community needs and communities supports (e.g., family, friends, doulas, lactation consultants, massage therapists) as essential to perinatal health care. One example of this is the need for breastfeeding/chestfeeding support:

*“Canada needs to support pregnant and birthing people as well as provide support for lactation. We know ∼90+% of people want to breastfeed/chestfeed yet the access to services to support them within the public system is almost nonexistent. Lack of education of our health professionals has significant impact on perinatal mental health.”*

Another respondent said: “*Doulas are seeing what doctors are not. We are with clients for extended periods of time, in their homes, at all hours of the day and night. We see new parents in their most intimate moments, often unguarded moments, and many are struggling.”* Community support roles (including in-home support workers, those that provide childcare and/or light housework, and allied health care practitioners) need to be incorporated into community health care planning and, as such, their roles need to be funded and valued by the appropriate provincial/territorial government. A new family can benefit from supports from many angles.

A few respondents pointed to the need for one collaborative and national approach to perinatal care given the fragmented nature of care and community-based initiatives that have been successful. For instance, one respondent stated that even though their community has advocates of care, “*there should be extra support at that part of the spectrum but as a singular tower of support it is unmoored. While better than nothing it*”*s nowhere near enough.”*

Success in other communities was experienced prior to COVID-19 where:

*Our community had a nice model where our peer support groups were co-facilitated by a children's mental health specialist and one of our RN's. This facilitated a focus not just on maternal mental health but gentle messaging re: infant mental health, attachment, parenting etc. Our group also co-facilitated on alternate weeks by an “outreach worker” with Early Childhood Educator training who could recommend parent/baby groups to enhance parent/baby connection and peer support for the parent.”*

Another respondent discussed the possible loss of funding for current community programs that threaten the ability of families to access community-based supports:

*“This program has been running for 12 years. It has been very successful, and we received very favorable opinions and feedback from our clients. It is hugely disappointing that the future of this program is questionable at this time.”*

Whereas other respondents, who felt disconnected from services and programming, stated that some were aware of what some other providers were doing: “*They call to do an EDPS at 6 weeks, but I never hear about it…and I do one at 6 weeks but there is not a process to share that information or to streamline access to programs.”*

Other practitioners resorted to other mediums of recruitment and advocacy such as social media:

*“Our clinic has created a postpartum Facebook group to assist clients in staying connected, and I am a moderator of this group.”* Others state that phone connection was also beneficial “*Having access to people over the phone is helping my clients with transportation issues, but it's more difficult to make a solid connection with the provider.”*

Other communities have not been so successful; for instance, for those respondents in rural and remote areas, screening and diagnosis can present logistical nightmares for access:

*“I now work in a very high resource area, but I previously worked an hour away and there was nothing. We could send people to rapid access if they were having an acute emergency, but half the time they would tell us to refer to a different service that would not accept referrals because we were out of the geographic catchment area. It was a nightmare.”*

Others echoed this sentiment saying that they “*would love to facilitate a support program in our small town/rural community as it is a great distance for clients to travel.”* From these responses, it is clear that perinatal mental health services are insufficient and require comprehensive, multi-jurisdictional responses.

### 3.4. Theme three: policy and larger systematic gaps and concerns

Respondents highlighted that a systemic failure in health care leading to barriers to accessing services and mental health supports. For instance, many respondents referred to the barrier presented by patients need to have a primary care provider in order to access perinatal health care. One respondent described:

“*You need to be rostered with the Family Health Team to access lots of the available services, so families without a family doctor are vulnerable. Community Mental Health won't accept referrals directly from midwives which creates a delay in accessing care if there is no local GP [general practitioner].*”

For those experiencing a PMHD, access to immediate care can be crucial to the long-term well-being of birthing persons and children. These systemic barriers are exacerbated by the inadequacy of public-funded perinatal health services:

“*There are very limited resources for PMHD, and even fewer therapy resources that are publicly funded*.” *If patients and/or families can afford private care, there are still wait times associated: “…with that private wait times are 2–4 weeks, public wait times for therapy can be 1 year.”*

A lack of a cohesive approach to perinatal mental health care was indicated in the fragmented aspects of screening, education and tools needed for providers. One respondent summarized this best in stating:

“*More access to community-based support, specialized training for family docs and psychiatrists as well as community mental health clinicians is all needed. My clients hear inaccurate information about medication, PMHD symptoms (“it's just the baby blues”), breastfeeding, infant sleep, etc. from their health care providers. Prenatal visits are not screening for mental health issues, even when clients indicate an history of mental health issues, and there is little follow-up.*”

Many respondents described a lack of screening of perinatal persons using appropriate tools, misinformation, lack of provider training in perinatal mental health care. For example, one participant stated, “*Few clients receive any kind of mental health screening. When they approach their doctors, support is ineffective or non-existent.”*

Other respondents pointed to the large impact that inadequacies in perinatal mental healthcare have on overall perinatal care:

*“The hospital system is completely failing parents in the postpartum period. After discharge, there is little to no access to care for physical or mental health except for a single 6-week follow-up visit. Even clients who have concerns before six weeks are having trouble accessing answers and care. I have had clients told they did not even need to bother with the 6-week follow up visit - even clients who have had a surgical birth. I have had better follow up for day surgeries than most of my clients have after their births. Postpartum parents deserve regular PPMD screening at intervals throughout the first year.”*

Respondents also raised concerns for the lack of treatment, support, and collaboration within their community:

*As a childbirth educator, I am not generally heavily involved in helping clients find mental health care. However, having tried to access mental health services for a family member, I found that it was very difficult. I had to find referral forms myself to take to the family doctor, request the referral and then there was a long wait time. Essentially, only medication was offered, and the severity of the condition was not understood by the psychiatrist. There was no follow-up unless initiated by the patient. CBD was offered. I know this is an evidence-based practice, but I have grave concerns about how it is being implemented. It seems to me that there is an emphasis on services where there is little to no human contact. True wellbeing occurs in the context of relationships. Is it our goal that people be minimally functional, or that we help them move towards wellbeing and thriving? Perinatal mental health has a profound impact on the children of these parents. Creating meaningful interventions at this point could lead to significantly fewer mental health problems for the next generation.”*

The lack of connectivity and communication, as one respondent pointed out, results in “*overlapping, nonexistent and noncommunicating services. Time and resource wasting, and the perinatal population is not best served.”*

Respondents pointed to the inadequacies of Canada's government with communities having to seek out their own resources, solutions. This results in “*too many chasms of care during pregnancy and postpartum due to the “silos” that exist in our local region and province, so parents don't receive the continuity of care that they need to be well and thrive. “* Solutions to this not only include a national strategy that includes guidelines but also supports for more collaborative approaches to care: “*Increased collaboration within circle of care to include Health Babies, Healthy Children [community programs], and other supports.”* Another respondent stated.

*“There needs to be more connectivity between programs/providers so that those in the field are aware of each other and what everyone is offering. This would help build awareness of [spell out] PPMD challenges and allow those that work in the field access more resources and refer to each other as well. Basically- a safety net.”*

Overall, some of our respondents felt that the greatest impact felt by policy and systemic approaches would be “*greater dissemination of information to health care providers. Answering these questions, I realize how little I know about resources actually available.”* Most felt that funding was a large barrier and failure of the system stating:

“*Funding remains the issue for people who struggle with perinatal mental health. Agencies such Community Mental Health Association of Muskoka and Parry Sound provide the services stretching resources to respond to the needs as best as possible. Overall, there needs to be an increase in funding to not only respond to Perinatal Mental Health but to promote wellness and provide education. As well a National PMHD strategy would also address most issues.”*

## 4. Discussion

Of the responses collected from over 400 Canadian perinatal care providers, this study found that the most profound barrier to adequate perinatal mental health care is the lack of coordinated national guidelines for perinatal mental health care. Actions taken to mitigate barriers included policy- and community-led approaches such as those communities who have undertaken training targeting population-based needs or in British Columbia, where provincial guideline have been developed. However, the lack of c guidelines and best practices creates barriers to access, treatment, and follow-up for parents and their children.

### 4.1. BIPOC and marginalized communities' issues and concerns- the need for specialized considerations, approaches, and care

We found that access to PMHD care to be uneven with many groups identified by perinatal care providers as being marginalized by the current system. These groups included BIPOC and those historically marginalized persons. Given these gaps, others have shown that marginalized individuals experienced larger increases in suicidality (19). This is not new, Darwin and Greenfield state that many studies do not account for the variability in family forms, ethnic backgrounds and the variety of determinants being largely ignored in general data collection and policy formation (20). For example, Darwin and Greenfield state that number of 2SLGBTQIA + families who have babies is unclear because of poor data collection procedures and protocols and thus impacts the types and variability of services needed within communities (20).

Barriers to PMHD care experienced by persons in marginalized groups included ethnocentrism and racism, mental health stigma, provider-related barriers such as lack of knowledge about perinatal mental illness, pandemic related restrictions, structural barriers related to lack of available care in certain areas and factors like cost and unavailable childcare. Similarly, in a Canadian qualitative study of 45 perinatal women and their partners and perinatal health care providers (physicians, psychologists, health administrators, nurses, midwives; respondents identified barriers to accessing perinatal mental health treatment included cost, stigma, long waitlist times, and a lack of treatment providers (21). As Darwin and Greenfield point out (20), there is a substantial need to address the social determinants of health when devising policies around perinatal care for women, men and children. Additionally, Daoud et al. (22, 23) found that there were a variety of determinants in the experience of discrimination for marginalized members of the population and this depended on type of discrimination experienced; these include migration, citizenship status, and racial and ethnic group of origin, and others. This results in ensuring that there is room within a national approach for more tailored approaches based on a variety of factors such as culture of origin, first language spoken, age of mother at birth, and other factors. This could lend support to community-led initiatives based on identified needs and gaps of providers in one area.

### 4.2. Community-Based initiatives as a response to innovate and move forward

It has been established that the rates of PMHD have increased during the COVID-19 pandemic (9, 24). Our study identified that access to PMHD care treatment has been negatively impacted by the COVID-19 pandemic as care providers identified that families were cut-off from their usual social supports from kin and friends, decreased opportunities for in-person care provider screening for PMHD, and that funding for PMHD programs was reduced. Hermann et al. (25) found the conversion of perinatal outpatient clinics, partial hospitals, and day programs to telehealth for consultation, medication management, and individual and group psychotherapy to have largely been successful despite regulatory, legal, and economic challenges associated with transitioning to virtual care. This suggests that while virtual platforms for care are necessary and may even be preferable in non-pandemic conditions, there is still a need to implement approaches that ensure perinatal persons are provided for in-person delivery options also. Taken together, the availability of different modalities of screening and treatment (virtual and in-person) are imperative for addressing PMHD.

Access to evidence-based perinatal mental health care that is relevant to the times and needs of clients is critical to improving family outcomes (15). PMHD can be associated with impaired functioning, especially in relation to a birthing person's ability to care for the infant and the formation of secure infant attachment, which may in turn be associated with poorer social, cognitive, and behavioural outcomes in the child (26, 27). It is therefore imperative that birthing persons (and their families) experiencing psychosocial problems and mental health conditions in the perinatal period are identified early to receive the timely support and care they need.

Collaborative care was voiced as being one of the most prominent areas needing to be addressed by a national strategy or best practice guidelines, in addition to training. There are many exemplars and evidence internationally showing the impact of collaborative care/multidisciplinary approaches on perinatal mental health outcomes (28). For example, psychiatric mother and baby units (MBU) provide specialist in-patient care for mothers who experience severe mental illness without separation from their baby; these units provide significant improvements in mental health and mother–infant interaction and are preferred by patients as they enable women to be co-admitted full-time with their babies, rather than being separated from them, as they would be if admitted to a general psychiatric ward. This type of approach may be warranted given the gaps identified. Furthermore, multidisciplinary teams on MBUs are trained in the treatment of perinatal mental health problems, and in childcare and development (29, 30). Additionally, patient-centered care is a vital component to this collaborative model and the treatment of postpartum mental health care (31).

Illustrating the importance of a collaborative approach to PMHD care, Webber and Benedict (28) stress that an interdisciplinary approach to screening and implementation of therapy for breastfeeding support and perinatal mental health is critical. For parents experiencing perinatal mental health problems, there is a demonstrated reciprocal relationship between breastfeeding difficulties and postpartum depression, and this highlights the importance of a multidisciplinary approach to caring for women experiencing PMHD.

Collaborative care may also take the form of collaboration between perinatal specialists and non-specialists. For Lever et al. (32) this may include specialist supports from physicians, midwives, and obstetricians/gynecologists paired with peer supports, nurse practitioners, doulas, lactation consultants, social workers, and the likes. In their interviews of women experiencing perinatal mental health, women valued both supports in their recovery process citing the personal care received from non-specialist forms or those who did not specialize in childbirth and maternal health. Miller et al. (33, 34) also cited that collaborative perinatal care results in higher rates of improvement in screening, diagnosis, and treatment overall.

#### 4.2.1. Policy and systematic failures: funding for perinatal mental health services programs

Often, funding for perinatal mental health falls under the umbrella of general mental health funding. This, however, is problematic as clear mandates are necessary to ensure that perinatal mental health programs receive sustained, focused funding. For example, within the Canadian context, provinces and territories administer and deliver most of Canada's health care services, with all insurance plans expected to meet national principles set out under the Canada Health Act. Services are funded with the assistance from federal cash and tax transfers, but specific mandates are generally determined by the province or territory (35). Few jurisdictions even have perinatal mental health on their radar (2). Targeted perinatal mental health care funding allocated to each province and territory to administer perinatal mental health programs is vital.

In contrast, Australia, Ireland, and Scotland have all announced dedicated funding for perinatal care (36–38). The Centre for Perinatal Excellence (39) located in Australia announced that it has allocated over $36 million to develop and implement digital tools for coordinating perinatal mental health. The iCOPE is an application that houses screening and diagnostic information and comes preloaded onto tablet formats, for ease of use within patient care settings. The data inputted allows for a range of services, pathways, and care to be established while also allowing for clinical-based reporting mechanisms, and longitudinal data collection and analysis (39).

Most of the national strategies published and implemented in Australia, Ireland, and Scotland have all included the required training, tools, and guidelines needed for providers of perinatal mental health. Even treatment options are weighed out in these guidelines. For example, Australia's COPE guidelines offer best practices with regards to establishing and maintaining the therapeutic relationship, considerations in cultural safety, components of trauma-informed care, considerations prior to screening and diagnosis, tools for each, assessing psychosocial factors using tools and other methods, and implementation considerations (39).

### 4.3. Limitations and strengths

These findings must be interpreted with caution because of design related limitations. First, because this was an analysis of textual responses to open-ended questions from an anonymous cross-sectional online survey, we were not able to follow up with participants to probe for further information or clarifications which limited the depth of the elicited data. Second, not all respondents provided textual responses to the open-ended questions, therefore the themes identified may not reflect all participants’ perceptions and experiences. As such, we cannot claim that data saturation was achieved. Notable strengths of this study include a large and diverse sample of perinatal care providers across Canada and fair representation of care providers who are visible minorities.

## 5. Conclusion

This study set out to understand the experiences of Canadian providers in perinatal service delivery. Over 400 respondents responded to our survey and after performing a qualitative analysis, three clear themes emerged from the responses: gaps in care for marginalized groups, gaps and supports in community settings, and policy and larger systemic considerations. When asked about services in the area and other information that is pertinent for the Canadian Perinatal Mental Health Collaborative to know in recommending policy approaches, themes around BIPOC and minority considerations and community approaches in addressing perinatal mental health were prominent in responses. Most of the respondents in our survey voiced the need for clear training, policy, and guidelines to address the variability in family forms and population needs. It is therefore vital that any perinatal mental health policy in Canada incorporate the experiences of the diverse Canadian population – geographically, racially, culturally, gendered, and otherwise – and that support for both families and health care providers include the allied health community as well. Within community-led initiatives, many respondents voiced that there is a need to include resources such as lactation consultants, doulas, and other service providers to help in addressing perinatal care across the continuum of development.

System gaps could be mitigated with a national perinatal mental health strategy that includes clinical practice guidelines or standards of care. These policies would clearly direct primary care providers in evidence-based assessment, screening, diagnosis, and treatment options. Looking to some of those countries and regions who have already developed significant headway to a more inclusive and well-defined and supported perinatal program, for example the United Kingdom, Australia and part of the United States, Canada has significant work to do in outlining appropriate methods for screening, diagnosis, and support for parents in the postpartum period. As outlined in the CPMHC report (2), further training of health care and social service providers in screening mechanisms is required to move forward and based on the results of this study, further clarification in how these screening tools are to be used and when is required from a national level. A national approach to PMHD would help to align a multitude of facets as outlined in the CPMHC report (2) including a comprehensive curriculum that allows a variety of health care and social service providers, including doulas, lactation specialists, and other non-traditional services, access to appropriate education, guidelines and screenings for all families. Our study further confirms this need and approach to reduce the number of families who are being missed and provide support in a holistic manner.

## 6. Statement of inclusion

Not all people who experience pregnancy, birth, and parenting, and seek perinatal care, including mental health care, identify as a woman or mother. Recognizing the diversity of gender identities and expressions, we commit to making improvements in the use of gender-neutral or gender-inclusive language. At the same time, we acknowledge that “mother” and “women” are terms used when referencing studies that involved mothers and women as respondents or when directly quoting survey participants or people with lived experience who have themselves used those terms. In addition, many fathers and partners are not screened for or do not disclose perinatal mental health symptoms, largely due to stigma, and not accessing needed treatments – this is a problem that also needs to be addressed.

It is vital that in our efforts to improve perinatal mental health across Canada, we are inclusive regarding sexual and gender identity, and to those who experience marginalization and oppression, such as persons with disabilities, Black, Indigenous, and People of Colour (BIPOC).

## Data Availability

The raw data supporting the conclusions of this article will be made available by the authors, without undue reservation.
